# Enterovirus A71: virulence, antigenicity, and genetic evolution over the years

**DOI:** 10.1186/s12929-019-0574-1

**Published:** 2019-10-21

**Authors:** Sheng-Wen Huang, Dayna Cheng, Jen-Ren Wang

**Affiliations:** 1 National Mosquito-Borne Diseases Control Research Center, National Health Research Institutes, Tainan, Taiwan; 2 National Institute of Infectious Diseases and Vaccinology, National Health Research Institutes, Tainan, Taiwan; 3 Department of Medical Laboratory Science and Biotechnology, College of Medicine, National Cheng Kung University, Tainan, Taiwan; 4 Center of Infectious Disease and Signaling Research, National Cheng Kung University, One, University Road, Tainan, 701 Taiwan; 5 Department of Pathology, National Cheng Kung University Hospital, Tainan, Taiwan

**Keywords:** Enterovirus A71, Epidemiology, Evolution, Recombination, Virulence, Antigenicity

## Abstract

As a neurotropic virus, enterovirus A71 (EV-A71) emerge and remerge in the Asia-Pacific region since the 1990s, and has continuously been a threat to global public health, especially in children. Annually, EV-A71 results in hand-foot-and-mouth disease (HFMD) and occasionally causes severe neurological disease. Here we reviewed the global epidemiology and genotypic evolution of EV-A71 since 1997. The natural selection, mutation and recombination events observed in the genetic evolution were described. In addition, we have updated the antigenicity and virulence determinants that are known to date. Understanding EV-A71 epidemiology, genetic evolution, antigenicity, and virulence determinants can expand our insights of EV-A71 pathogenesis, which may benefit us in the future.

## Introduction

Enterovirus A71 (EV-A71) has caused various symptoms and diseases ranging from hand-foot-and-mouth disease (HFMD), herpangina, rashes, and diarrhea, to aseptic meningitis, pulmonary edema, acute flaccid paralysis (AFP), brainstem encephalitis and Guillain–Barré syndrome [1, 2]. Although EV-A71 infections are often asymptomatic, severe symptoms can also result in neurological disease and even death [2]. Following the first EV-A71 identified in 1969, EV-A71 has been circulating in the Asia-Pacific region such as Japan in the 1970s [3], Asia in the 1980s [4], and Malaysia and Taiwan in the 1990s [1, 5–8]. Since the 1990s, large-scale epidemics have been observed [9]. Since then, EV-A71 infections have caused mortality rates ranging from < 0.5–19% in Asia-Pacific countries [1, 10–14].

As a member of the genus *Enterovirus* and the family *Picornaviridae,* enterovirus A71 (EV-A71) is a non-enveloped positive single strand RNA virus, containing 7.4-kb long RNA with a large open reading frame (ORF) flanked by the 5′ and 3′ untranslated region (UTR) [15]. The 5′ UTR consists of stem-loop RNA structures I to VI, which forms the internal ribosome binding site (IRES). Through the use of cap-independent translation mechanism, 5′ UTR is involved in viral protein translation and RNA replication [16, 17]. The large ORF is translated into a single polyprotein that is further cleaved into P1, P2, and P3 regions by viral proteases. The 2A protease (2A^pro^) of poliovirus can cleave the P1 capsid protein from the polyprotein. In contrast, the 3CD protease cleaved the P1, P2, and P3 precursors [18]. The mature structural proteins can be used in virus assembly, and non-structural proteins for replication, apoptosis induction, innate immunity repression and in the shutting down of host cell translation (reviewed in [19]). The P1 region encodes the capsid proteins VP1 to VP4. The VP1, VP2, VP3, and VP4 proteins form a symmetrical icosahedral structure. VP1, VP2, and VP3 are exposed on the external surface of viral capsid, whereas VP1 is the highest exposed protein among the capsids [20–22]. VP4, however, is the smallest of the P1 proteins and arranged within the icosahedral lattice. The structural protein VP1 contains the primary binding residues to two identified EV-A71 receptors, P-selectin glycoprotein ligand-1 (PSGL-1) and scavenger receptor B2 (SCARB2) [23, 24]. Tyrosine sulfation in the N-terminal region of PSGL-1 facilitates EV-A71 viral entry and replication in leukocytes, thus affecting viral replication [25]. PSGL-1 alone was found to be insufficient in enhancing EV-A71 infection in mice [26], while SCARB2 was found to be sufficient in causing neurological diseases in mice due to its roles in viral attachment, internalization, and uncoating [27]. In addition, some of the residues such as VP1–98, − 145, and − 164 [28], are antigenic sites for antibody recognition. The remaining P2 and P3 regions contain the non-structural proteins 2A to 2C and 3A to 3D, respectively. 3C^pro^ can inhibit retinoic acid-inducible gene I (RIG-I) -mediated interferon response [29]. Also, 3C^pro^ assists in the interaction of 5′ UTR with RNA-dependent RNA polymerase (RdRp) (3D^pol^) [29]. The RdRp lacks proofreading abilities, thus resulting in the high mutation rates of RNA viruses [30]. Since the amino acid changes might contribute to the alternation of protein properties, the substitutions within the viral genome may affect the viral protein interactions and replication. Here we focus on the review of the epidemiology, genetic evolution, antigenicity, and virulence determinants in EV-A71 viruses.

### Epidemiology of EV-A71

The first EV-A71 isolate was identified in 1969 in the United States [31]. During the 1970s, several outbreaks of EV-A71 with HFMD were reported in the USA, Australia, Japan, Hungary, Sweden, France, and Bulgaria [3, 31–37]. In the 1980s, outbreaks occurred in Asia, Brazil, Netherlands, and USA, but the viral activity reduced after these outbreaks [4, 38–42]. Starting from 1997, a large wave of EV-A71 activity appeared, causing HFMD around the Asia-Pacific region (Table 1). The first large HFMD and herpangina outbreak occurred in 1997 and further outbreaks appeared in 2000, 2003, and 2005 in Malaysia [6, 43]. In 1998, a large EV-A71 outbreak in Taiwan occurred, and increasing EV-A71 fatal cases were reported in 1999, 2000, 2001, 2004, 2005, 2008, and 2012 [44–47]. HFMD outbreaks were reported in Japan in the years 1984, 1987, 1990, 1997, 2000, and 2003, with the largest outbreak in 2003 [48]. Singapore had HFMD outbreaks in 2000, 2006, and 2008, with 2008 being the largest outbreak that Singapore had ever experienced [49, 50]. China only had sporadic reported cases of EV-A71 before 2004. After 2004, EV-A71 began to spread to the middle and northern regions of China, leading to an outbreak of HFMD in 2008 and in 2012 [12, 51, 52]. Large outbreaks of HFMD caused by EV-A71 were also reported in other countries such as Perth, Australia in 1999 [53], Vietnam in 2005, 2010–2011, 2012–2013, and 2016 [54, 55], Thailand in 2008–2009, 2011, and 2017 [14, 56, 57], and the Netherlands in 2007 [40]. These data suggest that the virus continued to circulate in the Asia-Pacific region, and had gradually spread to other countries.

**Table 1:** EV-A71 genotype changes in endemic countries from 1997 to 2018

	Australia	Austria	China	Japan	Korea	Malaysia	Netherlands	Norway	Singapore	Taiwan	UK	Thailand	Vietnam
1997	B3		C2	B3,B4,**C2**		**B3**,B4,C1,C2	C1,C2		B3,B4,C1				
1998	B3, C2		C4	C2		B4,C1			B3,C1	B3,B4,**C2**,C4	C1		
1999	**B3**^ab^**,** C2		C4	C2		B4,C1	C2		B3	B4,C2	C1,C2		
2000	B4, C1		C4	**B4**,C2	C3	**B4**,C1	C2		**B4**,B5,C1	**B4**	C1		
2001	B4,C1	C1	C4	C2	C3	B4,C1	C1		B4,C1	**B4**,C4	C1		
2002	C1	C1	C2,C4	B4,C2,C4	C3	B4,C1	C1,C2	C1	B4,C1	B4,C4	C1		
2003	C1,C4	C1,C4	C2,C4	B4,**B5**,**C4**	C3,C4	B4,**B5**,C1		C1	B4,B5,C1	B4,B5,C2			
2004	C4	C4	C2,C4	C4		B5,C1	C1,C2		B5	C2,**C4**	C1		
2005			C2,C4	C4	C3	B5,C1	C1,C2		B5	C2,**C4**,C5			C1,C4,**C5**
2006			C2,C4	C4		B5			**B5**	B5,C2,C4	C1,C2		
2007			C2,C4	C2,C4	C4	B5	C1,**C2**		B5	B5,C5			
2008			A,C2,**C4**	C2	C4	B5	C2		**B5**,C2	**B5**,C4,C5		B5,C1,C2,**C4**	
2009	C2, C4		C2,C4	C2	C2,C4	B5,C1			B4,B5,C1	B5,C1		C1,**C4**	
2010	B3, C2		C2,C4	C2	C4	B5				C4		B5	C5
2011			C4		C4	**B5**				B5,C4		B5	C4,C5
2012			**C4**	B5		B5				**B5**		**B5,**C4	B5,C4,C5
2013			C4	B5								B5	C4,C5
2014			C4										B5,C4,C5
2015													
2016				C4									C4
2017				B5								**B5**	
2018				B5									

^a^Predominant genotype which resulted in an outbreak

^b^Predominant genotypes were bold and underlined

### Genetic evolution

Due to the error-prone RdRp, RNA viruses generate 10^− 4^ to 10^− 6^ mutations per nucleotide [58], which leads to high mutation rates and increased genetic diversity [59]. Genetic evolution of EV-A71 can be clustered into three main genotypes A, B and C, whereas genotypes B and C include five sub-genotypes, B1-B5 and C1-C5, respectively [60]. EV-A71 has been documented in several studies and observed inter- and intra-genotype shifts in the evolution around Asian-Pacific countries (Table 1). The shifts had also co-occurred with EV-A71 outbreaks.

**Fig. 1:**
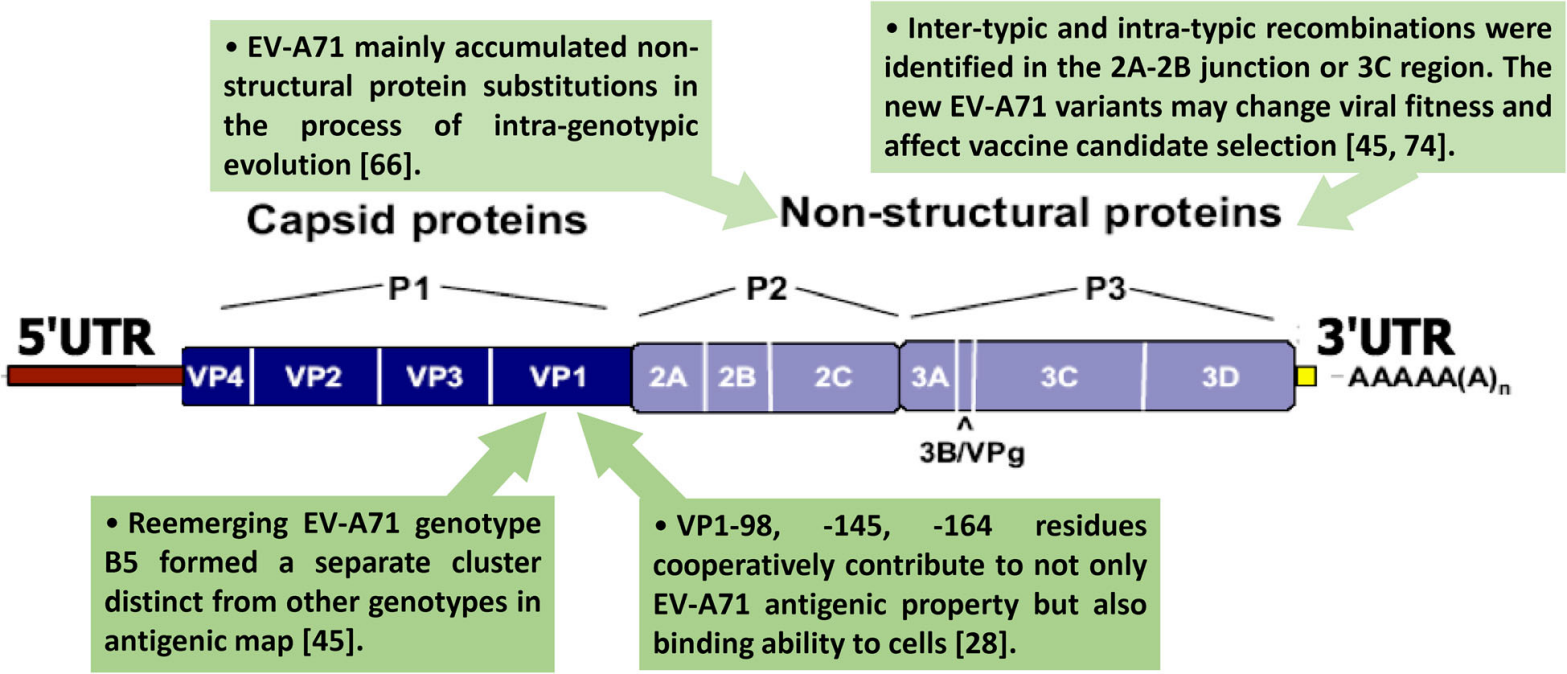
Genetic and antigenicity of EV-A71. Summary of genetic and antigenic determinants of EV-A71 throughout the viral genome that were reported

Analyzing the genotypic changes according to the recent evolutionary studies reports, inter-genotype shifts appeared in Taiwan and Japan. The predominant strain of the 1998 outbreak in Taiwan was EV-A71 genotype C2 with 90% having recombination with coxsackievirus A8 (CV-A8) and the remaining 10% were genotype B4 isolates [45, 61]. The genotype B4 isolates from the 1998 outbreak had similar sequences to those of the 2000 outbreak. In the 2000 and 2001 outbreak in Taiwan, the predominant strain was genotype B4, thus showing an inter-genotype shift from C2 to B4 [8, 62]. The predominant genotype in 2004 and 2008 outbreaks changed from B4 to C4 and from C4 to B5, respectively. The same order of genotype shifts was observed in Japan with genotypes C2, B4, C4, and B5 (Table 1) [63, 64]. Contrary to inter-genotypic evolution, intra-genotypic evolution occurred in China. EV-A71 genotype C4, which was circulating in China, had shown the continuous evolution of the virus through non-outbreak years (2004–2007) to the outbreak years (2008–2012) [65]. In China, genotype C4 had persisted through time, showing annual increase in the accumulation of non-structural protein substitutions. Continuous accumulation of amino acid substitution within the same genotype C4 through non-outbreak to the outbreak period might explain why the same genotype activity dramatically increased after 2008 in China. Similar accumulation of substitutions especially in non-structural protein region has been reported among the genotype B5 in Taiwan. Genotype B5 had also been found to accumulate evolutionary amino acid substitutions in non-structural proteins, thus causing a re-emergent outbreak in Taiwan in 2012 in the following of the same genotype outbreak in 2008 (Fig. 1) [66].

Recombination of EV-A71 viruses is another common phenomenon. In poliovirus, high nucleotide sequence identities within a region, mainly seen in P2 and P3, of parental strains are in favor of homologous recombination via a ‘copy-choice’ mechanism, resulting in a possible combination that may favor survival during the natural selection process [67, 68]. According to a study done by Woodman *et. al.,* recombination was found to be a replicative process that is RdRp-mediated [69]. Both intra- and inter-typic recombination can be found in EV-A71 viruses. Complete EV-A71 genomes were sequenced and phylogenetically analyzed via swapping through the regions of the whole genome in order to analyze recombination events [70]. Recombination events were documented in countries such as Japan, Malaysia, Singapore, and China [7, 45, 71–75]. Inter-typic recombination was observed in 1997 isolates from an outbreak in Malaysia. Chan and AbuBakar had demonstrated that recombinations involving EV-A71 with CV-A16 [72], and EV-A71 genotype C4 isolates with genotype C2 and CV-A16/G10-like viruses were evident [71]. Inter-typic recombination was also seen in EV-A71 genotype C2 which was the major genotype in the 1998 outbreak in Taiwan and Japan [7, 45, 71]. During the EV-A71 genotype C2 outbreak in 1998, using bootscan analysis the virus sequence showed recombination between EV-A71 genotype C2 and coxsackievirus A8 [45, 76]. From the 2000–2001 outbreak, the predominant EV-A71 genotype was B4, however, recombination analysis by Huang *et. al.* showed that the sequence resulted in a recombination of genotypes B3 and B2 [45]. For the outbreak in 2004–2005, the predominant C4 showed recombination between genotypes C and B [76]. Within these three outbreaks, both intra- and inter-genic recombination can be seen. Inter- and intra-typic recombinations were observed in China in a 2008 outbreak caused by both EV-A71 and coxsackievirus A16 (CV-A16) [74]. Yip *et. al.* (2010) had observed recombination events at the 2A-2B junction in EV-A71 genotypes B with genotype C, and EV-A71 genotype B with CV-A16 strain G-10 in the 3C region of EV-A71 viruses, while CV-A16 strains were found to possess recombination at the 2A-2B junction between CV-A16 strain G-10 and EV-A71 genotype A [74]. Recombination of enteroviruses were also found in Central China in 2011–2012, where co-circulation of CV-A16 and EV-A71 genotype C4 was observed [77]. Woodman *et. al.* (2019) had developed a cell-based assay in order to observe recombination events of EV-A71 and found that recombination events were highest in C2 genotype followed by C4 then B5 [69]. Chen *et. al.* (2010) had previously reported on recombination breakpoints and recombination frequencies of EV-A71. Recombination breakpoint locations may vary depending on the strain and country, however, the 3D^pol^ coding region shown to have the highest frequency at which recombination occurs as a unit [75].

### Virulence determinants of EV-A71

For the last two decades, increasingly more effort has been placed in understanding EV-A71. Several virus virulence determinants have been identified for EV-A71. Since the generation of mouse-adapted EV-A71 [78], an amino acid change in VP1 position 145 was identified for the adapted virulence. The change was identified as glycine (G) changing to glutamic acid (E) [79, 80]. Huang *et. al.* (2012) had also found that VP1^Q145E^ enhances binding of EV-A71 to mouse neuroblastoma (Table 2) [86]. In a non-human primate model, VP1^145E^ is responsible for the development of viremia and neuropathogenesis [90]. Huang *et. al.* (2012) observed that, in cooperation with VP1^Q145E^, an amino acid change in VP2 at position 149 from lysine (K) to methionine (M) (VP2^K149M^) is associated with the increase of RNA accumulation, viral cytotoxicity and uncoating in mice neuronal cells, and an increase in mouse lethality in vivo [86]. In contrast, Chang *et. al.* (2012) had observed that an amino acid change from glutamic acid (E) to glutamine (Q) in VP1 position 145 (VP1^E145Q^) was found in more severe cases of EV-A71 infections [87]. In addition, a VP1^145G/Q^ mutation had been found to be associated with the virus’ ability to bind to the receptor PSGL-1 while VP1^145E^ was associated with the inability to bind to PSGL-1 [88]. In a study done by van der Sanden *et. al.*, (2018) they had found infectivity of the human airway organoids were EV-A71 strain-dependent. Coincidentally, the well-known position VP1–145 was also found to be a key determinant of infectivity of human airways. Van der Sanden *et. al.* had identified VP1^145Q^ as a key determinant of increased infectivity in human airway organoids. In addition, in the absence of VP1^145Q^, viruses with relatively high replication rates were found to have both VP1^98K^ and VP1^104D^ mutations in genotype C5 strains. Therefore, VP1^98K^ and VP1^104D^ may be potential infectivity markers in specific viral strains [89]. More recently, Huang *et. al.* (2017) had demonstrated the evolution of EV-A71 virus within a single autopsy case from the 1998 EV-A71 outbreak in Taiwan. EV-A71 viruses were isolated from various tissues and analyzed, thus showing the evolution of the virus within the host, as well as tissue tropism. They had identified a dominant haplotype switch from VP1-31D to VP1-31G, with VP1-31G being dominant in the central nervous system (CNS), indicating possible contribution to CNS invasion of the virus. The VP1^D31G^ mutation was also found to enhance EV-A71 entry into neuroblastoma, increase virus growth rate and fitness in human neuronal cells, and had a higher proportion in the virus population in fatal patients than in HFMD patients [83]. Similarly, Cordey *et. al.* (2012) had analyzed the EV-A71 genome from various tissues of an immunocompromised patient. They had found that the mutation VP1^L79R^, located in the BC loop region, plays a critical role in cell tropism and affects the viral binding ability and fitness in neuronal cells in vitro [84]. In another recent study, VP1^107A^ was found to regulate the maturation of EV-A71. Zhang *et. al.* had discovered that VP1^107A^ allowed greater flexibility of the VP1 BC loop and regulated the efficient cleavage of VP0, influencing maturation and viral uncoating, thus increasing viral replication [85]. Zaini *et. al.* (2012) found that a VP1^K244E^ mutation is critical in mouse adaptation and virulence [91]. Nishimura *et. al.* (2013) had also found that mutations at VP1–244 can abolish virus binding to PSGL-1, while mutations at VP1–242 can influence virus binding. They had also proposed that depending on the protein at VP1–145, by its influence to control the exposure of the side-chain VP1^244K^, VP1–145 can act as a switch which controls PSGL-1 binding [88].

**Table 2:** Reported virulence determinants

Region	Position/Factor	Observations	Ref
5′ UTR	158C	• Increases EV-A71 translation and virulence in mice	[81]
	272G, 488 U, 700A/U	• Associates with higher prevalence in severe cases of EV-A71	[82]
VP1	31G	• Enhances EV-A71 entry into neuroblastoma • Increases viral growth and fitness in human neuronal cells • May facilitate CNS infection in humans	[83]
	31D	• Enhances replication, infectivity, and fitness in colorectal cells • Increases virion stability	[83]
	L79R	• Confers advantage in viral binding ability and fitness in neuronal cells • Potential determinant of host-adaptation and neurovirulence in humans	[84]
	107A	• Regulates EV-A71 maturation via the efficient cleavage of VP0 precursor • Increases viral uncoating efficiency	[85]
	145	• Under positive selection	[45]
	Q145E	• Enhances binding of EV-A71 to mouse neuroblastoma • Increases viral binding and RNA accumulation of EV-A71 • Cooperates with VP2^K149M^ to increase mouse lethality in vivo	[86]
	E145Q	• Observed in more severe cases	[87]
	G145E	• Associates with increase in virulence in mice	[79], [80]
	145Q/G, 244 K	• Associates with virus binding ability to PSGL-1	[88]
	145G/Q/R, 164E	• Associates with higher prevalence in severe cases of EV-A71	[82]
	145Q	• Key determinant of increased infectivity in human airway organoids	[89]
	98 K, 104D	• Potential infectivity markers of human airway organoids	[89]
	145E, 98 K/E	• Responsible for the development of viremia and neuropathogenesis, and increases levels of cytokines in Cynomolgus monkey model	[90]
	K244E	• Associates with mouse adaptation and virulence	[91]
VP2	K149 M	• Increases RNA accumulation, viral toxicity, virulence, and uncoating in mouse neuronal cells • Cooperates with VP1^Q145E^ to increase infectivity and mouse lethality of EV-A71, and increases cytotoxicity in Neuro-2a cells	[86]
2A	68 K	• Associates with higher prevalence in severe cases of EV-A71	[82]
3C	Region	• Can hamper the host innate defense by selectively blocking type I IFN synthesis	[92]
	N69D	• Decreases viral replication and virulence in RD cells • Regulates 3C^pro^ enzyme activity in mammalian cells	[93]
3D	I251T	• Contributes to a strong temperature sensitivity • Decreases virulence in neonatal mice	[94]

The 5′ UTR^U158C^ was found to be associated with translation and virulence in mice [81]. Other 5′ UTR positions such as guanine at 272 (272G), uracil at 448 (448 U), and adenine/uracil at 700 (700A/U) have been found to be associated with higher prevalence in severe cases of EV-A71 [82]. Li *et. al.* had also reported on other mutations that were associated with higher prevalence in severe cases of EV-A71: VP1^145G/Q/R^, VP1^164E^, and 2A^68K^ [82]. Apart from the structural region of the viral genome, the non-structural region had also been found to play a role in the virulence of EV-A71. Amino acid substitutions in this region had been found to increase viral fitness (Fig. 1) [66]. The 3C region has been reported to be able to interfere with the host innate defense by selectively inhibiting the synthesis of type I interferon (IFN), and in 3D polymerase, an I251T mutation resulted in decreased virulence of MP4 (a mouse adapted strain of EV-A71) and can change the temperature sensitivity of the virus [92, 94]. Arita *et. al.* (2005) had also reported that temperature-sensitive mutants that are located in the 5′ UTR, 3D^pol^, and 3′ UTR can cause an attenuation in neurovirulence [95]. The 69th residue of 3C^pro^ has also been found to influence replication and virulence of EV-A71. A 3C^N69D^ mutation had shown to attenuate virulence by impacting the substrate-binding site and catalytic active site. This mutation had also diminished 3C^pro^ activity and its ability to shutoff host cell metabolism, inhibition of host cellular transcription and host immune system [93]. With being under constant selection pressure in hosts, mutations often aid viruses in surviving the different environments within a host. This is often seen in quasispecies whereby the viral population works in cooperation to adapt to adverse growth condition [96].

### Antigenicity

With the increase in mutations, a change in antigenicity can also occur in addition to viral virulence. The capsid proteins have long been a target in producing antibodies against EV-A71 for immune system recognition. VP1 capsid region has been found to possess many antigenic determinants and is considered to play an important role in characterizing antigenicity [97]. Neutralizing antibodies (NAbs) against EV-A71 have been suggested as one of the most important factors in limiting the severity of EV-A71 infections [98]. Yu *et. al.* (2000) had demonstrated that adult mice were resistant to multiple EV-A71 challenges, thus producing neutralizing antibodies post-infection that play a role in limiting severity of EV-A71 infection. These neutralizing antibodies showed a protective role against EV-A71 by administering hyperimmune serum (1:128) 1-day post-infection in a mouse model. The anti EV-A71 NAbs was found to be able to effectively protect neonatal mice when passively immunized and when delivered and fed by an immunized dam [98]. Cross-neutralization activity of EV-A71 were also observed among various genotypes using guinea pig and rabbit antisera [99, 100]. Mizuta *et. al.* observed that guinea pig antisera against genotypes B2 and C1 had higher neutralization titers against genotypes B2, B4, and B5 but lower titers against genotypes A, C1, C2, and C4. Similarly, van der Sanden *et. al.* had similar results with rabbit antisera against genotypes B2 with higher neutralizing titers against genotypes B1 and B2 but lower titers against genotypes C1, C2, and A. However, rabbit sera against genotype C1 showed higher neutralization activity with EV-A71 genotypes A, B, and C [100]. Huang *et*. *al*. had previously investigated the cross reactivity and antigenic property of human antiserum from EV-A71 infected patients from 1998 to 2008 against human pathogenic EV-A71. Using sero-microneutralization data, an antigenic map was constructed which showed the antigenic diversity of the different genotypes. Based on this map, they had found that genotype B1 and B4 viruses were clustered closely together, genotype C2 and C4 formed a separate cluster from genotype B that was more spread out. Genotype B5, however, had formed its own cluster within the map (Fig. 1) [45]. These data suggest the difference in antigenic properties and the antigenic diversity among the various genotypes of EV-A71. Recently, Huang *et. al.* (2015) had confirmed that the amino acid residues VP1–98, − 145, and − 164 worked in a cooperative manner as antigenic determinants for B4 and B5 strains (Fig. 1). By creating reverse-genetics EV-A71 viruses containing mutations at VP1–98 K, VP1–145Q, and VP1-164E, they had found that these mutants significantly decreased neutralizing titers by 4-fold against the antisera of 3 of the 6 healthy individuals [28]. However, it was also noted that none of the single mutation alone was responsible for the antigenic changes, but rather all 3 mutations cooperatively influence the viral antigenic phenotype. With a combination of genotypic shifts, antigenic changes, as well as recombination events, EV-A71 may possess many traits that allow for the virus to continuously persist and escape herd immunity. These factors would thus aid in the event of another outbreak.

## Conclusions

Genotype shifts, changes in antigenic properties, and recombination events have shown to contribute to the evolution of EV-A71. Although there only genotype C4 available EV-A71 vaccine from China to date, increasing knowledge of the virus will better aid in the development of a vaccine that is able to protect against the different genotypes. Therefore, continuous surveillance of EV-A71 is required in order to better understand its epidemiology and viral evolution.

## Data Availability

Not applicable

## Abbreviations

2A^pro^: 2A protease
3C^pro^: 3C protease
3D^pol^: 3D polymerase
AFP: Acute flaccid paralysis
CNS: Central nervous system
CV-A16: Coxsackievirus A16
CV-A8: Coxsackievirus A8
EV-A71: Enterovirus A71
HFMD: Hand-foot-and-mouth disease
IFN: Interferon
IRES: Internal ribosome entry site
NAbs: Neutralizing antibodies
ORF: Open reading frame
PSGL-1: P-selectin glycoprotein ligand-1
RdRp: RNA-dependent RNA-polymerase
RIG-I: Retinoic acid-inducible gene I
RNA: Ribonucleic acid
SCARB2: Scavenger receptor B2
UTR: Untranslated Region
